# Shared response to changes in drainage basin: Phylogeography of the Yunnan small narrow‐mouthed frog, *Glyphoglossus yunnanensis* (Anura: Microhylidae)

**DOI:** 10.1002/ece3.6011

**Published:** 2020-01-18

**Authors:** Dong‐Ru Zhang, Hong Hui, Guo‐Hua Yu, Xin‐Qiang Song, Shuo Liu, Si‐Qi Yuan, Heng Xiao, Ding‐Qi Rao

**Affiliations:** ^1^ College of Life Sciences Yunnan University Kunming China; ^2^ State Key Laboratory of Genetic Resources and Evolution Kunming Institute of Zoology Chinese Academy of Sciences Kunming China; ^3^ College of Life Sciences Guangxi Normal University Guilin China; ^4^ Yingjing Administration of Daxiangling Nature Reserve Yaan China; ^5^ Kunming Natural History Museum of Zoology Kunming Institute of Zoology Chinese Academy of Sciences Kunming China; ^6^ Bioengineering College Sichuan University of Science and Engineering Yibin China

**Keywords:** drainage history, genetic structure, *Glyphoglossus yunnanensis*, phylogeography, secondary contact, shared response, southwest China

## Abstract

**Aim:**

With the late Cenozoic uplift of the Qinghai–Tibetan Plateau (QTP), drainage of the southeastern edge of the QTP changed significantly. However, the impact of this dramatic change on the geographical distribution and genetic diversity of endemic organisms is still poorly understood. Here, we examined the geographical patterns of genetic variation in the Yunnan small narrow‐mouthed frog, *Glyphoglossus yunnanensis* (Microhylidae), and two alternative hypotheses were tested: That is, the geographical distribution of genetic variation was determined by either the contemporary drainage basin or historical drainage basins.

**Location:**

The Mountains of southwest China.

**Materials and methods:**

Analyses were based on 417 specimens collected from across the distribution of the species. We reconstructed the genealogy (Bayesian and maximum parsimony methods) and assessed demographic history based on DNA sequencing data from mitochondrial and nuclear markers. We also mapped the genetic diversity and estimated the divergence times by a relaxed clock model.

**Results:**

The species has maintained a relatively stable population size without recent population expansion. Four major maternal lineages were identified with good support, one representing a possible cryptic species and the other three showing further subdivision. The distribution of these deeply differentiated lineages/sublineages corresponded well to geographical regions. The secondary contact zones and phylogeographic breaks in distinct lineages of *G. yunnanensis* were almost concordant with those of *Nanorana yunnanensis*.

**Main conclusions:**

Lineage division conformed to the hypothesis of drainage system evolution, that is, the phylogeographic pattern of *G. yunnanensis* was shaped by historical drainage patterns. Concordance in phylogeographic patterns may suggest a shared response to common hydrogeological history and also might indicate that there was more contribution of the drainage history than ecological or life‐history traits in structuring genetic variation between these two disparate codistributed taxa *G. yunnanensis* and *N. yunnanensis*.

## Graphical abstract

Lineage division conformed to the hypothesis of drainage system evolution, that is, the phylogeographic pattern of *G. yunnanensis* was shaped by historical drainage patterns. Concordance in phylogeographic patterns from different taxa *G. yunnanensis* and *N. yunnanensis* may suggest a shared response to common hydrogeological history.

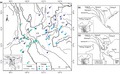

## 1 INTRODUCTION

The southeastern edge of the Qinghai–Tibetan Plateau (QTP) is an orogenetically young region that encompasses two geologically dynamic and biodiverse areas, namely the Hengduan Mountain and Indo‐Burma hot spots (Myers, 2003; Myers, Mittermeier, Mittermeier, da Fonseca, & Kent, 2000; Tang, Wang, Zheng, & Fang, 2006). These mountainous regions of southwest China and the rearrangement of their major river drainage systems are considered evolutionarily important (Stewart, Lister, Barnes, & Dalen, 2010). The Yunnan–Guizhou Plateau (YGP) formed a planation surface (or peneplain) with smooth relief during the middle–late Pliocene (Cheng, Liu, Gao, Tang, & Yue, 2001; He & He, 1993; He, He, & Zhu, 1985). With the strong uplift of the QTP, the Yunnan planation surface began to collapse from the late Pliocene to early Pleistocene (He et al., 1985). Along with the obvious change in topography, the previous lake‐centered drainage network was broken. In the late early Pleistocene, the most significant drainage system changes occurred on the Central Yunnan Plateau (CYP) and in northwest Yunnan (Cheng et al., 2001; Clark et al., 2004).

Increasing evidence has shown that the rearrangement of drainage systems has played a key role in shaping current geographical patterns of genetic and species diversity of aquatic organisms in many parts of the world (Adamson, Hurwood, & Mather, 2012; Burridge, Craw, & Waters, 2006; de Bruyn et al., 2013; Hurwood & Hughes, 1998; Kozak, Blaine, & Larson, 2006; Thomaz, Malabarba, Bonatto, & Knowles, 2015). The historical drainage systems in the southwest mountains of China are markedly different from present systems (Brookfield, 1998; Clark et al., 2004). Prior to the most recent uplift of the QTP, the initial drainage system along the plateau margin primarily consisted of tributaries of a single, southward flowing paleo‐Red River. With uplift of the QTP, disruption of the paleo‐drainage system occurred by river capture and reversal. The Jinsha, Yalong, and Dadu rivers connected to form the modern Jinsha River, which redirects drainage away from the southward Red River eastward into the East China Sea, and the Upper Mekong and Upper Salween rivers drained into their modern drainage positions. The impact of paleo‐drainage rearrangement on species diversification and geographical distribution has been evaluated via molecular phylogenetic studies among freshwater fish (Guo, He, & Zhang, 2005; He & Chen, 2006; Peng, Ho, Zhang, & He, 2006; Rüber, Britz, Kullander, & Zardoya, 2004) and a few riparian plant species and amphibians (Wang, Mao, Zhao, & Wang, 2013; Yan et al., 2013; Zhang, Chen, et al., 2010; Zhang, Comes, & Sun, 2011).

The potential for diversification triggered by environmental changes or climatic oscillations likely varies among taxa. Organisms with different ecological preferences may have differing responses to the same event, as well as contrasting evolutionary histories. Previous research on freshwater fish has provided evidence that changes in ancient drainage systems contributed to allopatric speciation and population range expansion (Guo et al., 2005; He & Chen, 2006; Peng et al., 2006; Rüber et al., 2004). Furthermore, phylogeographic studies on riparian plant species endemic to the hot–dry river valleys of the eastern Sino‐Himalayan region have indicated that current genetic structures were historically sculpted by paleo‐drainage patterns (Yue, Chen, Sun, & Sun, 2012; Zhang et al., 2011; Zhang & Sun, 2011). In contrast, however, recent research on pine species endemic to the major river valleys of southwest China has indicated that spatial genetic structure is a reflection of current geography and environmental factors rather than hydrogeological history (Wang, Mao, Zhao, & Wang, 2013).

The two patterns mentioned above have also been observed in amphibians from southwest China. For example, the current population structure of the Yunnan spiny frog, *Nanorana yunnanensis*, was primarily shaped by the historical drainage system (Zhang, Chen, et al., 2010). Studies on other amphibians from the same region have revealed that present‐day genetic structures were shaped predominantly by Pleistocene climatic oscillations (Li, Yu, Rao, & Yang, 2012; Yu, Zhang, Rao, & Yang, 2013), with deep river valleys identified as strong geographical barriers to dispersal (Li, Chen, Tu, & Fu, 2009; Yuan et al., 2016; Zhang, Rao, Rao, Yang, Yu, & Wilkinson, 2010). Differentiation in ecological adaptations among taxa has also been proposed to explain variation in the geographical patterns of genetic diversity. For example, the genetic structures of species living in lentic environments, for example, Yunnan pond frog (*Babina pleuraden*) (Li et al., 2012) and Red Knobby Newt (*Tylototriton shanjing*) (Yu et al., 2013), differ from those inhabiting lotic environments, for example, *N. yunnanensis* (Zhang, Chen, et al., 2010). Drainage system evolution may have significant influence on the genetic structure of species preferring lotic water (Yu et al., 2013). However, *Leptobrachium ailaonicum* (Zhang, Rao, et al., 2010) occupies montane streams similar to *N. yunnanensis* (Zhang, Chen, et al., 2010), but both have highly distinct phylogeographic patterns. Thus, the responses of organisms to changes in drainage patterns are complex. Further research is needed to explore the impact of hydrogeological history of drainage basins on the geographical distribution and genetic structure of endemic taxa in southwest China.

*Glyphoglossus yunnanensis* occurs in southwest China (Yunnan, southern Sichuan, and western Guizhou), adjacent northern Vietnam, and presumably in adjacent Laos (Frost, 2019). It inhabits moist and soft soil, and breeds in puddles of rainwater and ditches at elevations of 1,700–3,600 m (personal observation). Its distribution overlaps considerably with *N. yunnanensis* and *B. pleuraden*. Vicariance theory assumes that common genealogical distribution patterns and geographical position of lineage breaks across multiple organisms in the same geographical range may originate from shared biogeographical history (Rissler & Smith, 2010). Thus, *G. yunnanensis* is an ideal species for exploring the mechanisms driving the phylogeographic patterns of species in this region from a comparative phylogeographic perspective.

Our objective was to examine the impacts of drainage system rearrangement on the genetic patterns of *G. yunnanensis*. We tested two alternative hypotheses: That is, geographical distribution of the genetic variation of *G. yunnanensis* was determined primarily by the contemporary drainage basin or by the historical drainage basins. We collected samples from throughout the distribution range of *G. yunnanensis*. Using DNA sequence data from both mitochondrial and nuclear genes, we analyzed genealogical and population genetics of the nucleotide sequence data. If the geographical structure of the genetic variation was best explained by the contemporary drainage basin, we would expect haplotypes from the same drainage basins have closer phylogeographic relationships and cluster together. On the other hand, phylogeographic breaks would be expected among haplotypes sampled from the same drainage basins if historical drainage basins were responsible for the phylogeographic structure.

## 2 MATERIALS AND METHODS

### 2.1 Sampling

**Figure 1:**
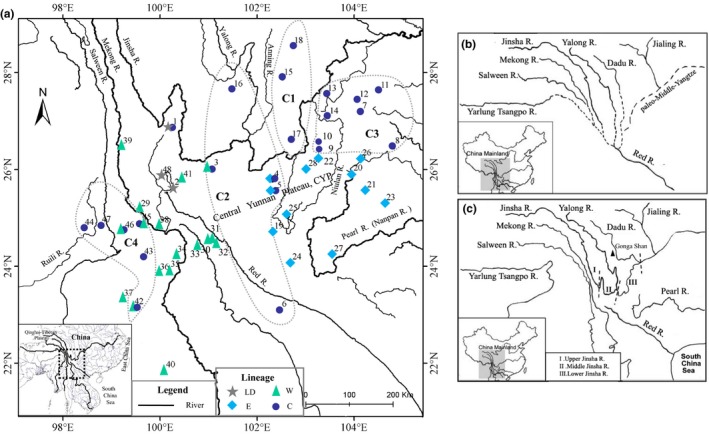
Information on sampling localities and hydrological history. (a) Geographical distribution of sampling populations of *G. yunnanensis*. Localities are numbered as in Supporting Information Table S1 in Appendix S1. Solid triangles, circles, diamonds, and five‐pointed star correspond to major matrilines (lineages W, C, E, and LD, respectively) in Figure 2. Overlap of different symbols denotes sympatric occurrence of distinct clades. Grayish dotted line designates geographical distributions of four main haplotype subclades of clade C in Figure 2. Both (b) and (c) are adapted from T. C. Zhang et al. (2011). Paleo‐drainage pattern prior to major river reversal/capture (b) and modern river pattern after putative capture and reversal events (c) are shown, respectively

From 2013 to 2016, a total of 417 individuals were collected from 48 localities throughout the distribution range of *G. yunnanensis*, except Vietnam (Supporting Information Table S1 in Appendix S1; Figure 1). Sample size ranged from 5 to 20 individuals per locality, depending on population density. Tissues, including toe clips and muscle and liver samples, were collected and preserved in 95% ethanol and frozen at −20°C in the laboratory. Voucher specimens were deposited at the Kunming Institute of Zoology, Chinese Academy of Sciences. Other members of the genus *Glyphoglossus* and several related species were chosen as hierarchical outgroup taxa for genealogical reconstruction (Supporting Information Table S2 in Appendix S1) according to studies of Das, Min, Hsu, Hertwig, and Haas (2014), Peloso et al. (2016), and Matsui et al. (2011).

### 2.2 Laboratory procedures

Using the proteinase K/sodium dodecyl sulfate (SDS) method (Sambrook & Russell, 2001), genomic DNA was extracted from tissue samples. Partial segments of two mitochondrial genes, *cytochrome oxidase I* (COI) and *cytochrome b* (CYTB), were amplified for all individuals. Mitochondrial ribosomal subunit *16S* rRNA and nuclear gene *tyrosinase* (Tyr) were amplified only for a small subset of individuals (176 and 58 samples, respectively) belonging to different lineages identified by preliminary analysis. References for primers are given in Supporting Information Table S3 in Appendix S1. For the mitochondrial gene COI, due to difficulty in polymerase chain reaction (PCR) amplification and sequencing for some samples, primer H‐t COI (Stuart & Parham, 2004) was used to pair with primer Chmf4 in this study.

All amplifications were conducted in 25 μl volume reactions and were initiated at 95°C for 5 min followed by 35 cycles at 94°C for 1 min, 46°C–55°C for 1 min, 72°C for 1 min, and a single final extension at 72°C for 10 min. Standard annealing temperatures are given in Supporting Information Table S3 in Appendix S1. Negative controls were run for all amplifications. The PCR products were purified with a Gel Extraction Kit (Tsingke Co., Ltd., Beijing, China). Cycle sequencing reactions were performed using a BigDye Terminator Cycle Sequencing Kit (v2.0, Applied Biosystems, USA), and sequencing was conducted on an ABI PRISM 3730XL automatic DNA sequencer (Applied Biosystems, USA) with both forward and reverse primers.

Nuclear gene clonal sequencing was carried out for individuals that contained more than one ambiguous site and failed to infer alleles using PHASE v2.1.1 (Stephens & Scheet, 2005; Stephens, Smith, & Donnelly, 2001). First, the nuclear gene Tyr fragment was re‐amplified following the above procedures. The purified PCR products were then cloned into a pClone007 Versatile Simple Vector Kit (Tsingke Co., Ltd., Beijing, China) and transferred into Trelief^TM^ 5α chemically competent cells (Tsingke Co., Ltd., Beijing, China). Plasmids carrying the PCR fragment were then extracted and sequenced. For each PCR product, at least four clones were randomly selected, with one then sequenced.

All newly derived sequences have been deposited on GenBank under the accession numbers MN851304–MN852232 and MN860291–MN860466.

### 2.3 DNA sequence alignment

DNA sequences were assembled and edited using DNASTAR 5.0 in Lasergene v.7.1.0, aligned using ClustalX 1.81 (Thompson, Gibson, Plewniak, Jeanmougin, & Higgins, 1997) with default parameters, and then examined and revised by eye in MEGA 7.0 (Kumar, Stecher, & Tamura, 2016). Protein‐coding nucleotide sequences were translated to amino acids to confirm alignment. Allele sequences of individuals with heterozygous sites were inferred using PHASE 2.1.1, with the algorithm applied five times with different seeds and the assumption of the stepwise mutation mechanism for multiallelic loci relaxed. The input files for PHASE were generated using the web tool SEQPHASE (Flot, 2010). Individuals whose haplotype analysis failed underwent clonal sequencing. The final nuclear gene dataset was the combination of both haplotype estimates and clonal sequencing.

### 2.4 Datasets

Three datasets were available under study. Two mtDNA datasets were prepared for different analyses. DATA I, for genealogical reconstruction, were the concatenation of *16S* rRNA, COI, and CYTB, and contained 195 individuals plus seven outgroup taxa. DATA II, which excluded individuals representing the cryptic species based on genealogical analysis, were the combination of COI and CYTB of 406 samples for genetic analysis. Identical haplotypes were generated using DnaSP 5.10 (Librado & Rozas, 2009). Nuclear gene was the third dataset.

### 2.5 Genealogical reconstruction and divergence dating estimation

To estimate phylogenetic relationships among mitochondrial haplotypes, Bayesian inference (BI) and maximum parsimony (MP) were implemented using DATA I. The best‐fit models of sequence evolution were determined using ModelTest 3.7 (Posada & Crandall, 1998). The BI analysis was performed using MrBayes 3.2.1 (Ronquist & Huelsenbeck, 2003) with six million generations, and two independent runs starting from different random trees were performed with four Markov chains. The chains were sampled every 100 generations. Convergence between the two runs was checked in Tracer 1.7.1 (Rambaut, Drummond, Xie, Baele, & Suchard, 2018). The first 25% of trees were discarded as burn‐in, and the remaining sampled trees were used to construct majority rule consensus trees and estimate Bayesian posterior probabilities (BPP) of the tree nodes. The MP analyses were conducted in PAUP4.0b10 (Swofford, 2002) using a heuristic search with 1 000 random‐addition sequence replicates, with support for nodes of the resulting MP tree assessed based on 1 000 bootstrap replicates.

Lineage divergence times were estimated in BEAST v1.8.4 (Drummond, Suchard, Xie, & Rambaut, 2012) implemented using the CIPRES Science Gateway portal (Miller et al., 2015). No fossil data were available to serve as internal calibration points within the genus *Glyphoglossus* or within the closely related genus *Microhyla*. Therefore, we assumed a substitution rate of 0.65%–1.00% (mean = 0.8%) per million years (Ma) for CYTB based on evolutionary rates commonly suggested for anurans (Macey et al., 1998, 2001; Monsen & Blouin, 2003). The mutation rate was multiplied by the ratio of the average distance for combined sequences versus that for CYTB alone, after which we deduced the substitution rate of the concatenated fragment (Qu et al., 2011). The net average distance (Da) was estimated to be 0.059 for combined gene fragments, 0.855 times greater than that of CYTB alone. The evolutionary rate of 0.65%–1.00% (mean = 0.80%) per Ma for CYTB was multiplied by a factor of 0.855 to deduce the substitution rate of all fragments combined, 0.556%–0.855% (mean = 0.684%). We employed an uncorrelated log‐normal relaxed molecular clock with constant‐size tree prior. Analyses were implemented for 100 million generations using the GTR model of nucleotide substitution with gamma‐distributed rate variation among sites. Effective sample size for each parameter and convergence were checked using Tracer v1.7.1.

The allele network of the nuclear gene Tyr was constructed. After generation of a neighbor‐joining (NJ) tree based on uncorrected *p*‐distances in MEGA 7.0, the network of haplotypes was visualized in Haploview (Salzburger, Ewing, & Von Haeseler, 2011).

### 2.6 Molecular diversity and genetic structure

Analysis of DATA II was conducted in ARLEQUIN 3.5 (Excoffier & Lischer, 2010). Haplotype diversity (*h*) and nucleotide diversity (π) were estimated for overall and for each population with more than five samples, respectively. Geographical partitioning of genetic diversity was inferred via two approaches. First, the *G. yunnanensis* populations were grouped using spatial analysis of molecular variation (SAMOVA) (Dupanloup, Schneider, & Excoffier, 2002) and were separately analyzed based on mtDNA and nuDNA data. Second, the genetic structure of the populations was inferred by an analysis of variance framework (analysis of molecular variance, AMOVA) (Excoffier, Smouse, & Quattro, 1992). There were three grouping options. First, all samples were divided into groups based on mtDNA lineages E, W, and C. Second, groups of populations were defined according to the contemporary drainage basin where the individual population resided: that is, eastward river basins (Jinsha and Pearl rivers) and southward drainage basins (Ruili, Salween, Mekong, and Red rivers). Third, due to the subdivisions within each lineage, all sampled populations were grouped based on sublineages. Population genetic differentiation was evaluated by pairwise values of *F*_ST_. Both AMOVA and *F*_ST_ used Kimura's two‐parameter (K2P) (Kimura, 1980) genetic distance.

### 2.7 Population demography

The demographic history of each main phylogenetic clade was investigated using Extended Bayesian Skyline Plot (EBSP) analyses (Drummond et al., 2012; Heled & Drummond, 2008) implemented in BEAST2 (Bouckaert et al., 2014), based on mtDNA DATA II (COI + CYTB, 1,140 bp) and nuDNA data (546 bp). The substitution rate (mean = 0.00738) of the mtDNA gene fragment was deduced as in previous BEAST analyses and used as a reference to guide rate estimates for the nuDNA. The analysis was set up following the recommendations of Heled (2015) for BEAST2. Chains were run for 100 million generations sampling every 10,000 steps, and three independent runs were conducted. Final graphs were generated following Heled (2010) with a burn‐in cutoff of 20%.

## 3 RESULTS

### 3.1 Sequence characteristics

No premature stop codons were observed in the mitochondrial protein‐coding genes, indicating that the sequences were obtained from functional genes rather than nuclear mitochondrial pseudogenes. For the ingroup, the *16S* rRNA, COI, and CYTB alignments were 1,014, 570, and 570 bp in length, respectively. For DATA I, of the 2,154‐bp nucleotides from 195 aligned ingroup individuals, there were 240 potentially phylogenetically informative sites and 119 generated haplotypes. For DATA II, 110 haplotypes were collapsed from 406 ingroup sequences that contained 128 potentially parsimony informative sites. Haplotypes were geographically restricted, 83.64% of them were private. No widespread haplotype was found.

For Tyr, a total of 58 individuals were sequenced. One to seven heterozygous sites were observed in some individuals. After allele estimates and clonal sequencing, the nuclear gene was 546 bp in length, and 71 haplotypes were generated from 101 allele sequences that included 23 parsimony informative sites.

### 3.2 Genealogical reconstruction and divergence time estimation

**Figure 2:**
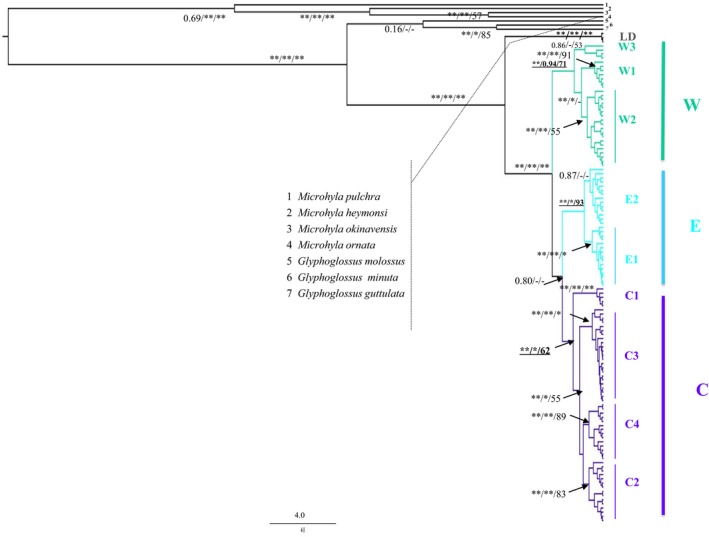
Bayesian inference (BI) tree estimated using BEAST based on mitochondrial DATA I. Posterior probabilities (BEAST), BI postprobabilities, and maximum parsimony (MP) bootstrap values (*>95, **>99%) are shown for main clades. Vertical bars show lineage/sublineage assignment

GTR + I + G was selected as the best evolutionary substitution model. The BI, MP, and BEAST trees consistently revealed that the ingroup contained four highly supported major lineages and that these independent evolutionary lineages showed strong geographical structure: that is, LD, W (western), E (eastern), and C (central) (Figures 1 and 2). Lineage LD included haplotypes only from populations 1–2, and 48, and was sister to the group of the other three lineages. Lineage W contained three sublineages (i.e., W1, W2, and W3) and consisted of haplotypes from western and southwestern areas of Yunnan (populations 29–41), where drainages are tributaries of the Mekong, Red, Salween, and Jinsha rivers. Lineage E was comprised of haplotypes from the eastern areas of Yunnan (populations 19–28), where drainages are tributaries of the Jinsha and Pearl rivers. Sublineage E1, which contained haplotypes from localities 19, 24, 25, and 28, was highly supported. Lineage C included four well‐supported sublineages, with C1 sister to the clade consisting of other three sublineages with strong support. C1 was distributed in southwestern Sichuan (populations 15, 17, and 18), C2 was located in central Yunnan (populations 3–6 and 16), and C3 was composed of haplotypes from northeast Yunnan and northwest Guizhou and Lijiang (populations 1 and 7–14). Drainages in these regions belong to tributaries of the Jinsha River and several from the Red River. Sublineage C4 occurred in a small area of western Yunnan (populations 42–47), where drainages are tributaries of the Salween, Mekong, and Ruili rivers.

Lineages C and E co‐occurred in populations 4 and 5 (Figure 1). Lineages W and C were sympatric in sites 3, 42, and 45–46 (Figure 1). Net average genetic distances (Kimura‐2 parameter model) between lineages were W/C, 1.4%; W/E, 1.5%; and C/E, 1.3%. Lineage LD was deeply separated from the other three lineages: that is, LD/E, 9.6%; LD/W, 9.1%; and LD/C, 9.2%.

**Figure 3:**
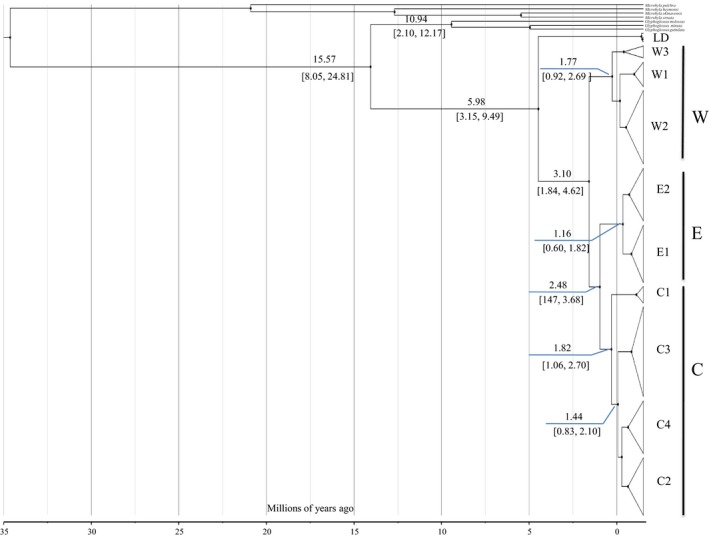
BEAST time estimation for *G. yunnanensis*. Branch lengths are proportional to divergence times. Matrilines correspond to Figure 2. Numbers at nodes are average ages. Numbers in bracket on nodes are 95% confidence intervals

Lineage LD diverged from the remaining ingroups about 5.98 Ma (95% HPD, 3.15–9.49 Ma). Lineage W diverged about 3.10 Ma (late Pliocene) (95% HPD, 1.84–4.62 Ma), and lineage E diverged from lineage C about 2.48 Ma (95% HPD, 1.47–3.68 Ma). The most recent common ancestor of lineage W was 1.77 Ma (early Pleistocene) (95% HPD, 0.92–2.69 Ma), with lineages E1–E2 diverging about 1.16 Ma (early Pleistocene) (95% HPD, 0.60–1.82 Ma). In lineage C, lineage C1 first diverged about 1.82 Ma (early Pleistocene) (95% HPD, 1.06–2.70 Ma), whereas lineages C2–C4 became isolated from each other about 1.44 Ma (95% HPD, 0.83–2.10 Ma) (Figure 3).

**Figure 4:**
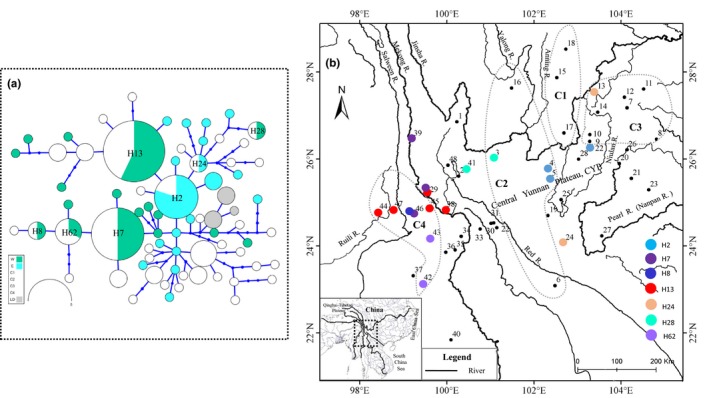
(a)Median‐joining network of nuclear gene Tyr. Circle size is proportional to relative number of individuals sharing a particular allele, and circle color represents an individual's membership in clades/subclades from maternal analysis. (b) Geographical distribution of main shared alleles. The localities number and grayish dotted line are the same as Figure 1. The overlap of solid circles of different colors indicates the co‐occurrence of different alleles

Compared with the maternal pattern, the network of Tyr loci (Figure 4) did not recover most lineages strongly supported by the mitochondrial gene tree, except for lineage LD. Moreover, most of the alleles were private, while shared alleles were mainly found among some populations in the border zone of different mitochondrial clades, for example, H7, H8, H13, and H62 were shared among populations in the border zone between lineages W and C4; H28 shared by W/C2, and H2 and H24 by E/C. No alleles were shared between lineages W and E.

### 3.3 Population genetic diversity

Overall *h* and π were 0.9831 ± 0.0014 and 0.023105 ± 0.011225, respectively, and varied considerably among populations (Supporting Information Table S1 in Appendix S1). Populations 3–5, 17, 42, and 45–46, where different lineages or sublineages co‐occurred and alleles were shared, had remarkably higher π.

### 3.4 Population genetic structure

The SAMOVA results revealed similar significant *F*_CT_ values for different numbers (K) of groups (from three to nine) (Table 1). For the mtDNA data, the *F*_CT_ value reached a plateau at K = 6, but the six suggested groups did not completely correspond to mtDNA lineages (Figure 2). Localities 3–4, 6, 16–17, and 42–47, geographically corresponding to sublineages C2 and C4, were grouped together (Supporting Information Figure S1 in Appendix S1). Furthermore, the eastern region included two groups: that is, 5, 19, and 24–25 grouped to form E1, and 20–23 and 26–28 grouped to form E2. With the increase in K value, *F*_CT_ also increased, but to a lesser extent, and new subdivision emerged (Supporting Information Figure S1 in Appendix S1), which were roughly consistent with mtDNA lineages. Unlike mtDNA analyses, SAMOVA based on nuDNA data suggested no phylogeographical division (Table 1).

**Table 1:** Results of SAMOVA and AMOVA for grouping populations. All *p* < .01

Grouping option	mtDNA						nuDNA		
	%Among groups	%Among populations within groups	%Within populations	*F*_SC_	*F*_ST_	*F*_CT_	*F*_SC_	*F*_ST_	*F*_CT_
3	50.2	31.65	18.15	0.63552	0.81849	0.5020	0.30335	0.59527	0.41904
4	54.12	27.2	18.69	0.59275	0.81314	0.54116	0.26115	0.56201	0.40721
5	58.17	22.11	19.72	0.52866	0.80283	0.58169	0.24345	0.55283	0.40893
6	60.1	19.88	20.02	0.49826	0.79978	0.60095	0.22989	0.52531	0.38361
7	61.94	17.84	20.23	0.4686	0.79774	0.61939	0.22455	0.51857	0.37916
8	64.37	14.92	20.71	0.41873	0.79291	0.64373	0.23506	0.50608	0.3543
9	66.01	13.11	20.89	0.38554	0.79112	0.66006	0.23483	0.49524	0.34033
Three mtDNA lineages	59.82	31.1	9.08	0.7741	0.90924	0.59823			
Six mtDNA lineages	71.86	19.03	9.11	0.67614	0.90885	0.71855			
Six contemporary drainage basins	30.33	48.93	20.73	0.70238	0.79265	0.30331			

The AMOVA results revealed significant geographical structure in the mtDNA genetic variation at all hierarchical levels examined (*p* < .01; Table 1). When populations were grouped according to the mtDNA lineages (six strongly supported clades W, E, and C1–C4 by three methods) (Figure 2), AMOVA produced the maximum *F*_CT_ value (0.7186). In contrast, grouping of contemporary drainage basins did not adequately explain the distribution of genetic diversity across the study (*F*_CT_ = 0.3033).

Pairwise *F*_ST_ estimates ranged from 0.00 to 1.00, and analysis of genetic differentiations showed strong evidence for correspondence between geographical area and lineage as well as between geographical subdivision and sublineage. Genetic differentiations between geographical zones and between subdivisions were significantly high (Table 2). In contrast, *F*_ST_ estimates within geographical zones and subdivisions were lower. Therefore, the mtDNA of *G. yunnanensis* demonstrated considerable geographical structure.

**Table 2:** Pairwise *F*_ST_ among geographical zones/subdivisions (*p* < .05), and summary of average pairwise *F*_ST_ values within geographical zones/subdivisions

	*F*_ST_		*F*_ST_
Between W and C	0.8048*	Within C	0.6206
Between W and E	0.8194*	Within W	0.5051
Between C and E	0.8168*	Within E	0.4235
Between C1 and C2	0.7615*	Within C1	0.1225
Between C1 and C3	0.9355*	Within C2	0.2893
Between C1 and C4	0.8946*	Within C3	0.4346
Between C2 and C3	0.6587*	Within C4	0.3549
Between C2 and C4	0.6068*	Within W1	0.0913
Between C3 and C4	0.8091*	Within W2	0.4493
Between W1 and W2	0.4918*	Within W3	0.2053
Between W1 and W3	0.6059*	Within E1	0.1058
Between W2 and W3	0.8171*	Within E2	0.3833
Between E1 and E2	0.5772*		

* *p* < .05

### 3.5 Population historical demography

**Figure 5:**
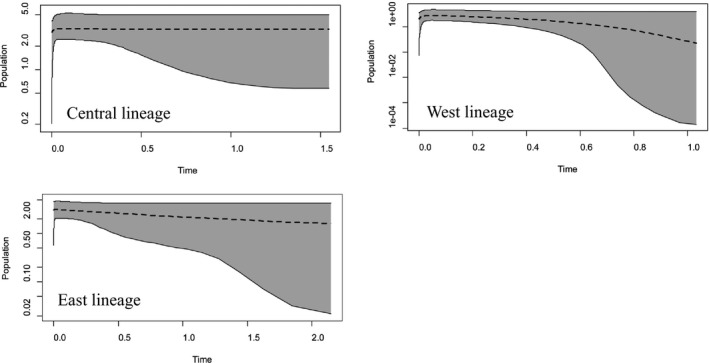
Demographic reconstructions with the Extended Bayesian Skyline Plot (EBSP) for the Central, West, and East lineages. They are based on 52, 22, and 22 samples sequenced of nuclear and 185, 122, and 99 samples sequenced of mtDNA, respectively. Dashed line: mean population size. Shaded area: 95% confidence interval. *Y*‐axis is in log scale, and *X*‐axis is time before present (million years, Ma)

Historical dynamic analyses for main lineages yielded similar results (Figure 5), with no sign of population size change in their recent history.

## 4 DISCUSSION

### 4.1 Genetic structure and historical drainage pattern

The matrilineal genealogy (Figure 2) revealed clear genetic divergence (W, C, and E) in *G. yunnanensis*, and further division occurred in each main clade. The analysis of mitochondrial variation by SAMOVA and AMOVA supported this division (Table 1; Supporting Information Figure S1 in Appendix S1). However, our nuDNA data did not find the pattern identified in mtDNA, possibly due to incomplete lineage sorting or implied gene flow. So, we largely restricted this section discussion to the present mtDNA data as they provided a finer geographical resolution compared with the nuDNA analysis.

The geographical distribution of *G. yunnanensis* exhibits greatest similarity to the montane stream amphibian species *N. yunnanensis*, and the Yunnan pond frog *B. pleuraden*. In contrast to montane stream species, however, *G. yunnanensis* breeds and lays eggs in still water, mainly in the Jinsha River basin. This geographical distribution highlights its use as an ideal model organism for testing scenarios regarding the role of historical drainage rearrangement on species genetic diversity. We may expect its genetic structure to be unaffected by drainage basin history, such as that found for the Yunnan pond frog (*B. pleuraden*) (Li et al., 2012) and red knobby newt (*T. shanjing*) (Yu et al., 2013), two other standing‐water taxa. To prove this conjecture, we hypothesized that a strong association would exist between the geographical distribution of genetic variation and contemporary drainage basins (H0) and that historical drainage basins would be accountable for the phylogeographic structure (H1). H0 would be rejected by evidence suggesting a phylogeographic break in the genetic relationships of haplotypes from the same drainage basin. Individuals from Jinsha River basin were deeply divergent and scattered in different mtDNA lineages (Figures 1 and 2; see the details below). The AMOVA results (Table 1) showed that differentiation among populations within the same drainage was almost twice that between different drainages (48.93% and 30.33%, respectively) when groupings were based on the current drainage system. These lines of evidence showed there was a phylogeographic discontinuity in the genetic variation within the same drainage basin. Thus, we rejected H0 and supported that contemporary drainage basins are connected by historically independent drainage systems. Groupings based on the mtDNA lineages, which conformed to the hypothesis of drainage system evolution, explained the greatest amount of variation (Table 1) and gave strong support to H1.

Deep phylogeographical divergence among populations was found in the modern Jinsha River drainage basin. Haplotypes from some tributaries (including populations 19, 22, 25, and 28; lineage E; Figures 1 and 2) demonstrated deep divergence from those of other tributaries (populations 1, 3–5, and 7–18, lineage C; population 41, lineage W). This discovery was predicted by the scenario that the modern Jinsha River drainage basin developed from a connection of historically independent paleo‐drainage basins (Clark et al., 2004; Li, Yang, Huang, Ge, & Wang, 2009; Ren, Yang, & Han, 2006) (Figure 1). The geographical transition zone between lineages C and E is roughly concordant with the CYP, which divided the ancient Red, Pearl, and Jinsha rivers (Wang & Wang, 2005) (Figure 1). The divergence time of *G. yunnanensis* (~3.10 Ma) roughly corresponds to the late Cenozoic extrusion uplift of the CYP (2 ~ 4 Ma) (Wang & Wang, 2005), which is proposed to be associated with the rapid uplift of the QTP in the same period (Cheng et al., 2001; Clark et al., 2004; Yang, Li, Huang, & Ge, 2010). This scenario is further supported by phylogeographic studies on endemic amphibian species and riparian plant species in this region (Zhang, Chen, et al., 2010; Zhang et al., 2011; Zhang & Sun, 2011).

The above hypothesis is also supported by the presence of three geographically distinct haplogroups (sublineage C1, C2, and C3) in the Jinsha River drainage basin (Figures 1 and 2). Sublineage C1 mainly consists of haplotypes from three populations 15, 17–18 distributed in the Lower Jinsha River. This genetic distribution is likely along the paleo‐Dadu River and is concordant with the hypothesis that the paleo‐Dadu River was a tributary of the paleo‐Red River (Clark et al., 2004) (Figure 1b). The estimated divergence time between C1 and the other three subgroups (C2–C4) (~1.82 Ma) roughly corresponds to the early Pleistocene capture of the paleo‐Dadu/Anning River due to the uplift of the Gonga Shan region (Clark et al., 2004; Jiang, Wu, Xiao, & Zhao, 1999; Li & Ming, 2011), which resulted in significant changes from lacustrine to fluviatile sediment (Jiang et al., 1999). Sublineage C2 includes four populations from the Jinsha/Yalong River drainage basin (3–5 and 16) and one population (6) from the Red River drainage basin, which agrees with the hypothesis that the Jinsha and Yalong rivers once flowed southwards into the paleo‐Red River prior to the capture of the paleo‐Yalong River by the Lower Jinsha River (Clark et al., 2004) (Figure 1b). These genetic patterns have also been revealed in other endemic species (Li et al., 2012; Yue, Chen, Sun, & Sun, 2012; Zhang, Chen, et al., 2010; Zhang & Sun, 2011).

The C3 sublineage mostly occurs in the Lower Jinsha River, but also shows a scattered distribution in the Middle Jinsha River (population 1) (Figure 1). The sharing of some haplotypes among these disjunctive populations (populations 1 vs. 7–14) is difficult to explain, but similar patterns have also been found in riparian plant species such as *Terminalia franchetii* (Zhang et al., 2011). Sublineage C3 also consists of populations from Niulan River, and their close genetic relationship is concordant with the hypothesis that the Niulan River–Yongshan–Xinshizhen paleo‐drainage was a north‐flowing tributary (Li & Ming, 2011; Li, Yang, et al., 2009), different from the south‐flowing Yalong and Dadu rivers.

Since the Pliocene, with the exception of the upper reaches in northwest Yunnan, the drainage basins on both sides of the Red River are of antiquity and have experienced little change relative to other regions (Cheng, Chen, Luo, & Peng, 1994; Cheng et al., 2001; Clark et al., 2004), thus resulting in higher genetic homogeneity (Li et al., 2012; Zhang, Chen, et al., 2010; Zhang, Rao, et al., 2010). That is, we would expect populations within the same drainage system are more closely related. Populations from east of Yunnan and from west of the Red River respectively clustered together and formed Lineage E and lineage W (Figure 1). Lineage C4 includes haplotypes from both the Ruili and Salween River basins (Figure 1). The patterns are in accordance with the hypothesis.

C4 is distributed geographically far from the other sublineages of group C, but genetically closer to C2 (Table 2). The close genetic relationship between these geographically isolated sublineages (C1–C4) indicates a probable connection or no obvious dispersal barriers among these regions during the Pliocene, which coincides with the hypothesis that the YGP formed a planation surface with smooth relief during the middle–late Pliocene (Cheng et al., 2001; He & He, 1993; He et al., 1985). Several phylogeographic studies of endemic species also confirm this inference (Wang et al., 2013; Zhang, Chen, et al., 2010; Zhang et al., 2011). However, more samples from areas between the sublineages are needed to clarify the issue.

The phylogeographical structure of *G. yunnanensis* substantially corresponds with historical drainage patterns and was largely in concordance with codistributed but disparate Yunnan spiny frog (*N. yunnanensis*, Zhang, Chen, et al., 2010). Expectation of the concordance of genetic structure among taxa with disparate traits is almost null in most comparative phylogeographic researches (Papadopoulou & Knowles, 2016). Thus, the congruence here might indicate that there was more contribution of the drainage history than ecological or life‐history traits in structuring genetic variation between these two disparate codistributed taxa. However, there are discordant phylogeographic patterns between codistributed Yunnan pond frog (*B. pleuraden*, Li et al., 2012) and *G. yunnanensis*. Thoroughly sampling and reanalysis of Yunnan pond frog would be helpful to clarify the discordance. In addition, other amphibian species studied in this area, such as Knobby Newt (*T. shanjing*, Yu et al., 2013) and Ailao Mustache Toad (*L. ailaonicum*, Zhang, Rao, et al., 2010), are mainly found west of the Red River. At this finer scale, broadly congruent structuring was identified among all amphibian species investigated here. To be sure, more phylogeographic data from endemic organisms with different ecological or life preferences are needed and require further comparative studies to gather a realistic overview of biological consequences of the historical drainage change.

### 4.2 Persistence during Pleistocene climatic oscillations

Cyclical Pleistocene glaciations have had profound influence on population dynamics (Hewitt, 2000, 2004), whose spatial effects depend on latitude and topography. Population demographic dynamics vary with life history and geography (Hewitt, 2004). Our study showed that *G. yunnanensis* diverged prior to Pleistocene climatic change and survived Pleistocene climate oscillations intact; thus, its mtDNA genetic diversity was highly structured geographically, as shown by the SAMOVA/AMOVA and *F*_ST_ results. Historical demographic analysis did not reveal any signs of recent population expansion, indicating that the species has maintained a relatively stable population size over time. Thus, based on the demographic stability coupled with low vagility, inference could be made that lineages have sufficient geographical stability to preserve the phylogenetic characteristics of ancient hydrological rearrangement and the geographical background of lineage splitting (Kozak et al., 2006). Much effort has been made to explore the pattern and extent of Pleistocene climatic oscillations on the geographical distribution and genetic diversity of organisms on the QTP and adjacent areas (Qiu, Fu, & Comes, 2011; Wan et al., 2018; Yan et al., 2013; Yang, Dong, & Lei, 2009; Yu et al., 2013; Yuan et al., 2016). Although complex topography is characterized by buffering effects of dramatic climatic oscillations (Muellner‐Riehl, 2019; Tzedakis, Lawson, Frogley, Hewitt, & Preece, 2002), responses to Pleistocene climatic changes vary among species in southwest China. Several studies have shown long‐term demographic stability (Fan et al., 2013; Wan et al., 2018; Yan et al., 2013), whereas others have revealed extensive population expansion (Liu et al., 2013; Yu et al., 2013; Zhan, Zheng, Wei, Bruford, & Jia, 2011; Zhang, Chen, et al., 2010; Zhang, Rao, et al., 2010). Yuan et al. (2016) believed that the ecological requirements of species within the same region, such as lowland and montane areas, should be considered in response to climate fluctuations. However, two montane species, *Quasipaa boulengeri* (Yan et al., 2013) and *N. yunnanensis* (Zhang, Chen, et al., 2010), exhibit completely distinct responses to Pleistocene climate fluctuations. In our opinion, ecological opportunity (Wagner, Harmon, & Seehausen, 2012) and microecological niches should also be included to further investigate the responses of organisms to Pleistocene climatic oscillations.

### 4.3 Secondary contact

Sympatric distribution of distinct lineages was demonstrated in a few populations (Figure 1; populations 3–5, 42, and 45–46), which may result from incomplete lineage sorting, admixture events, or both (Wendel & Doyle, 1998). If incomplete coalescence occurred, we would expect extensive coexistence of haplotypes from different lineages. Contrary to the incomplete coalescence, if secondary contact occurred, coexistence of haplotypes from different lineages would only be observed in populations where two distinct lineages were adjacent to each other, as observed in the present study (Figure 1). Furthermore, the higher π (Supporting Information Table S1 in Appendix S1) and absence of intermediate divergent haplotypes are suggestive of secondary contact. Although nuclear data from Tyr do not well‐resolve relationships among *G. yunnanensis*, they show some noteworthy features. Perhaps because of the small sample size of Tyr sequences, allele sharing is not common, and it is mainly distributed in the border zone between geographically adjacent matrilines (W/C4; W/C2; and E/C; Figure 4), roughly corresponding to the sympatric areas of different lineages. For example, allele H13 is shared by populations 29, 38, 44–45, and 47 from matrilines C4 and W. Allele H28 is present in populations 3 and 41 from matrilines C2 and W, and allele H2 was shared by E and C2. These patterns indicate either gene flow or the retention of ancestral polymorphisms. More samples and multiple informative nuclear loci, such as short tandem repeats (microsatellites), are required to distinguish between the possibilities.

Like the matriline patterns of *N. yunnanensis* (Zhang, Chen, et al., 2010), sympatric distribution of distinct lineages also occurred in areas adjacent to major drainage divides (Figure 1). For example, lineages C and E co‐occurred along the CYP, which forms the Jinsha, Red, and Pearl River watershed (Wang & Wang, 2005) (Figure 1 **)**. This further indicates that the evolution of drainage basins in southwest China has had significant impact on the contemporary geographical distribution of *G. yunnanensis* genetic variation.

## 5 IMPLICATIONS FOR CONSERVATION

Sympatric distribution of distinct lineages in *G. yunnanensis* and *N. yunnanensis* occurs in many areas. For example, in the CYP, localities 4, 5, and 17 from this study largely correspond to *N. yunnanensis* sample sites 32, 55–56, and 70 (Zhang, Chen, et al., 2010), which all harbor a particularly high level of nucleotide diversity. The same pattern also appears in western Yunnan for both species. These areas may be major genetic reservoirs for organisms and should be considered for priority protection, pending further monitoring and assessment of additional species.

## 6 CONCLUSIONS

Our study documents the genetic structure within *G. yunnanensis*. We found that historical drainage systems are responsible for the divergence between lineages/sublineages. The frog diverged prior to Pleistocene climatic change and experienced a stable demographic history thereafter. Sympatric distributions of distinct lineages resulted from secondary contact, and these areas are major genetic reservoirs and require priority protection. The phylogeographic patterns of *G. yunnanensis* are almost in concordance with those of *N. yunnanensis*, thus showing a shared response to hydrogeological history. Comparative phylogeographic studies of other endemic taxa in southwest China are now required to estimate whether their population genetic structures still preserve evidence of these late Cenozoic changes in palaeohydrology.

## CONFLICT OF INTEREST

None declared.

## AUTHOR CONTRIBUTIONS

DRZ, DQR, and HX conceived the ideas; HH and DRZ carried out fieldwork, with active assistance from XQS, SL, JSW, and SQY. DRZ performed laboratory and data analyses, and drafted the manuscript. GHY helped to revise the manuscript and gave constructive advice. All authors contributed to and approved the manuscript.

## Data Availability

All sequences were uploaded to GenBank. Additional supporting information may be found online in the Supporting Information section. Datasets for this study are available at: https://doi.org/10.5061/dryad.2z34tmph4
