# Supplementary material for: Shared response to changes in drainage basin: Phylogeography of the Yunnan small narrow‐mouthed frog, Glyphoglossus yunnanensis (Anura: Microhylidae)
Source: Ecol Evol. 2020 Jan 18;10(3):1567–80. doi: 10.1002/ece3.6011 (PMC7029061; doi:10.1002/ece3.6011)

**Supporting Information**

**Shared Response to Changes in Drainage Basin: Phylogeography of** **the Yunnan Small Narrow-Mouthed Frog,** ***Glyphoglossus yunnanensis* (****Anura: Microhylinae)**

Dong-Ru Zhang^1,2,#^, Hong Hui^2,#^, Guo-Hua Yu^3^, Xin-Qiang Song^4^, Shuo Liu^5^, Si-Qi Yuan^6^, Heng Xiao^1,*^ , Ding-Qi Rao^2,*^

**Appendix S** This appendix includes Tables S1 – S3 and Fig. S1.

**Table S1** Summary of sample site details for *Glyphoglossus yunnanensis*. For each population sampled, geographic origin, identified lineage or sublineage (W, C, E and C1–C4), sample size (N), coordinates (latitude, longitude), and contemporary drainage are given. Haplotype diversity (*h*) and nucleotide diversity (π) for each population with sample size ≥ 5 are presented.

| Label | Population origin | Lineage | N | Elevation | Latitude | Longitude | Nucleotide diversity | Gene diversity | Contemporary  drainage |
| --- | --- | --- | --- | --- | --- | --- | --- | --- | --- |
| 1 | YN, Lijiang | C3, LD | 14 | 2695 | 26°51'18.17″ | 100°13'39.9″ | 0.001662 +/-0.001156 | 0.8545 +/- 0.0657 | Jinsha River |
| 2 | YN, Dali | LD | 6 | NA | 25°36'23.35″ | 100°16'3.50″ | NA | NA | Mekong River |
| 3 | YN, Dayao, Santai | W, C2 | 8 | 2064 | 26°00'32.55″ | 101°04'46.76″ | 0.015979 +/- 0.009270 | 0.8095 +/- 0.1298 | Jinsha River |
| 4 | YN, Wuding, Chadian | C2, E | 12 | 2324 | 25°46′22.46″ | 102°19′13.19″ | 0.013252 +/- 0.007186 | 0.8788 +/- 0.0595 | Jinsha River |
| 5 | YN, Wuding, Shizishan | C2, E | 11 | 2281 | 25°32′30.89″ | 102°22′35.69″ | 0.015528 +/- 0.008438 | 0.7636 +/- 0.0833 | Jinsha River |
| 6 | YN, Lvchun, Gekui | C2 | 6 | 2576 | 23°05'23.99″ | 102°28'47.99″ | 0.003939 +/- 0.002605 | 0.8667 +/- 0.1291 | Red river |
| 7 | GZ, Weining, Xueshan | C3 | 20 | 2346 | 27°10'51.66″ | 104°08'05.88″ | 0.000883 +/- 0.000692 | 0.6842 +/- 0.0830 | Jinsha River |
| 8 | GZ, Shuicheng,Yushe | C3 | 7 | 2160 | 26°27'27.43″ | 104°48'43.26″ | 0.002009 +/- 0.001430 | 0.8095 +/- 0.1298 | Pearl river |
| 9 | YN, Huize, Jinzhong | C3 | 1 | 2000 | 26°24'22.23″ | 103°16'48.84″ | NA | NA | Jinsha River |
| 10 | YN, Huize, Wuxing | C3 | 1 | 2206 | 26°33'48.98″ | 103°16'55.67″ | NA | NA | Jinsha River |
| 11 | YN,Zhenxiong, Wanchang | C3 | 8 | 1559 | 27°36′29.39″ | 104°30′55.19″ | 0.000879 +/- 0.000749 | 0.4643 +/- 0.2000 | Jinsha River |
| 12 | YN, Yiliang, Longjie | C3 | 11 | 1904 | 27°25′16.92″ | 104°04′32.23″ | 0.002597 +/- 0.001658 | 0.6000 +/- 0.1539 | Jinsha River |
| 13 | YN, Zhaoyang, Sujiaxiang | C3 | 9 | 2784 | 27°32′41.30″ | 103°25′23.00″ | 0.001713 +/- 0.001212 | 0.3889 +/- 0.1644 | Jinsha River |
| 14 | YN, Ludian, Huodehong | C3 | 3 | 2267 | 27°04′32.40″ | 103°27′41.20″ | NA | NA | Jinsha River |
| 15 | SC, Zhaojue, Jiefangxiang | C1 | 13 | 3129 | 27°52′24.48″ | 102°31′18.51″ | 0.002077 +/- 0.001358 | 0.6538 +/- 0.1060 | Jinsha River |
| 16 | SC, Yanyuan, Baiwu | C2 | 10 | 2580 | 27°37′59.58″ | 101°29′03.28″ | 0.000000 +/- 0.000000 | 0.0000 +/- 0.0000 | Jinsha River |
| 17 | SC, Huidong, Lunan | C1, C2 | 8 | 2827 | 26°36′03.33″ | 102°41′05.92″ | 0.011254 +/- 0.006475 | 0.8571 +/- 0.1083 | Jinsha River |
| 18 | SC, Yuexi, Puxiong | C1 | 11 | 3083 | 28°31′36.76″ | 102°43′47.37″ | 0.000447 +/- 0.000460 | 0.5091 +/- 0.1008 | Jinsha River |
| 19 | YN, Anning,Bajie | E | 1 | 1993 | 24°42'03.25″ | 102°19'18.34″ | NA | NA | Jinsha River |
| 20 | YN, Zhanyi, Yanfang | E | 1 | 2114 | 25°53'51.40″ | 103°57'17.44″ | NA | NA | Pearl river |
| 21 | YN, Fuyuan, Mohong | E | 14 | 1868 | 25°33'13.36″ | 104°14'07.39″ | 0.003952 +/- 0.002323 | 0.8462 +/- 0.0512 | Pearl river |
| 22 | YN, Huize, Dahai | E | 15 | 3589 | 26°13'27.17″ | 103°15'41.35″ | 0.003495 +/- 0.002076 | 0.7048 +/- 0.0535 | Jinsha River |
| 23 | YN, Fuyuan, Huangnihe | E | 16 | 1712 | 25°17'23.58″ | 104°37'58.01″ | 0.005024 +/- 0.002845 | 0.4500 +/- 0.1507 | Pearl river |
| 24 | YN, Tonghai, Lishan | E | 8 | 2065 | 24°03′53.29″ | 102°41′50.51″ | 0.006034 +/- 0.003620 | 1.0000 +/- 0.0625 | Pearl river |
| 25 | YN, Kunming, Xishanqu | E | 16 | 2185 | 25°04′07.73″ | 102°37′12.73″ | 0.005085 +/- 0.002876 | 0.7417 +/- 0.0727 | Jinsha River |
| 26 | YN, Xuanwei, Dongshan | E | 4 | 2188 | 26°12′41.27″ | 104°08′14.13″ | NA | NA | Pearl river |
| 27 | YN, Mile, Dongshan | E | 14 | 2111 | 24°14′01.42″ | 103°33′20.10″ | 0.002419 +/- 0.001529 | 0.5934 +/- 0.1438 | Pearl river |
| 28 | YN, Dongchuan, Hongtudi | E | 1 | 2627 | 25°59'42.10″ | 103°01'33.79″ | NA | NA | Jinsha River |
| 29 | YN, Yongping, Shuixie | W | 1 | 1989 | 25°09′54.74″ | 99°34′31.76″ | NA | NA | Mekong River |
| 30 | YN, Jingdong, Taizhong | W | 11 | 2390 | 24°31′03.81″ | 101°00′46.87″ | 0.003364 +/- 0.002066 | 0.5455 +/- 0.0722 | Red river |
| 31 | YN, Chuxiong, Xishelu | W | 1 | 2358 | 24°31′54.05″ | 101°04′23.39″ | NA | NA | Red river |
| 32 | YN, Shuangbai, Ejia | W | 7 | 2355 | 24°25′12.74″ | 101°08′40.93″ | 0.002937 +/- 0.001959 | 0.4762 +/- 0.1713 | Red river |
| 33 | YN, Jingdong, Jingpin | W | 9 | 2250 | 24°23'21.48″ | 100°45'47.16″ | 0.002302 +/- 0.001537 | 0.4167 +/- 0.1907 | Red river |
| 34 | YN, Yunxian, Yongbao | W | 15 | 2268 | 24°13′15.60″ | 100°19′40.30″ | 0.004182 +/- 0.002428 | 0.5429 +/- 0.1327 | Mekong River |
| 35 | YN, Linxiang, Daxueshan | W | 6 | 2475 | 23°54′42.80″ | 100°11′15.10″ | 0.000000 +/- 0.000000 | 0.0000 +/- 0.0000 | Mekong River |
| 36 | YN, Linxiang, Boshang | W | 18 | 2001 | 23°51′20.30″ | 99°58′20.30″ | 0.000615 +/- 0.000544 | 0.3856 +/- 0.1280 | Salween River |
| 37 | YN, Cangyuan, Mengjiao | W | 10 | 2090 | 23°18′56.40″ | 99°13′35.30″ | 0.003252 +/- 0.002028 | 0.7778 +/- 0.0907 | Salween River |
| 38 | YN, Fengqing, Xinhua | W | 2 | 2300 | 24°49′35.80″ | 99°58′01.50″ | NA | NA | Mekong River |
| 39 | YN, Lanping, Yingpan | W | 5 | 2752 | 26°27'28.80″ | 99°12'21.60″ | 0.000000 +/- 0.000000 | 0.0000 +/- 0.0000 | Mekong River |
| 40 | YN, Menghai, Xiding | W | 11 | 1743 | 21°50'22.39″ | 100°05'41.41″ | 0.000575 +/- 0.000540 | 0.3273 +/- 0.1533 | Mekong River |
| 41 | YN, Dali, Binchuan | W | 1 | NA | 25°46′36.84″ | 100°26'58.06″ | NA | NA | Jinsha River |
| 42 | YN, Cangyuan, Danjia | W, C | 6 | 2188 | 23°08′31.20″ | 99°26′39.00″ | 0.019292 +/- 0.011487 | 0.8667 +/- 0.1291 | Salween River |
| 43 | YN, Yongde, Wumulong | C | 18 | 2591 | 24°10′34.80″ | 99°38′42.80″ | 0.001327 +/- 0.000935 | 0.5033 +/- 0.0639 | Salween River |
| 44 | YN, Lianghe, Xiaochang | C | 8 | 2071 | 24°46′31.50″ | 98°25′02.70″ | 0.000000 +/- 0.000000 | 0.0000 +/- 0.0000 | Irrawaddy River |
| 45 | YN, Changning, | C, W | 20 | 2086 | 24°52′56.01″ | 99°36′26.31″ | 0.019008 +/- 0.009774 | 0.7632 +/- 0.0595 | Mekong River |
| 46 | YN, Shidian, Xunyang | C, W | 16 | 2180 | 24°43′27.81″ | 99°14′56.97″ | 0.019066 +/- 0.009949 | 0.6750 +/- 0.0853 | Salween River |
| 47 | YN, Baoshan, Lujiang | C | 2 | 2215 | 24°50′01.94″ | 98°46′01.95″ | NA | NA | Salween River |
| 48 | YN, Dali, Cangshan | LD | 1 | 2900 | 25°52′05.58″ | 100°01′04.82″ | NA | NA | Mekong River |

YN, Yunnan Province; SC, Sichuan Province; GZ, Guizhou Province.

**Table S2** Outgroup species used in this study and corresponding accession numbers.

| **Species** | **Voucher** | **Locality data** | ***16S*** | **COI** | **CYTB** | **TYR** |
| --- | --- | --- | --- | --- | --- | --- |
| *Glyphoglossus minuta* | KUHE:52463 | Malaysia: Pahang, Temerloh | AB598340^2^ |  |  |  |
| *Glyphoglossus guttulata* | FMNH 252955 | Vietnam: Gia-Lai Province: Ankhe District | DQ283144^3^ | KM509768^4^ | Missing | DQ282937^3^ |
| *Glyphoglossus molossus* | CAS 210056 | Myanmar: Sagaing | KM509135^4^ | KM509798^4^ | AB201225^1^ | KM509928^4^ |
| *Microhyla_ornata* |  |  | NC_009422 | NC_009422 | NC_009422 |  |
| *Microhyla_okinavensis* |  |  | NC_010233^5^ | NC_010233^5^ | NC_010233^5^ |  |
| *Microhyla_heymonsi* |  |  | NC_006406^6^ | NC_006406^6^ | NC_006406^6^ |  |
| *Microhyla_pulchra* |  |  | KX021976 | KX021976 | KX021976 |  |

**Reference:**

1. Das, I., Min, P. Y., Hsu, W. W., Hertwig, S. T., & Haas, A. (2014). Red hot chili pepper. A new Calluella stoliczka, 1872 (Lissamphibia: Anura: Microhylidae) from Sarawak, East Malaysia (Borneo). *Zootaxa, 3785*, 550-560. doi:10.11646/zootaxa.3785.4.4

2. Matsui, M., Hamidy, A., Belabut, D. M., Ahmad, N., Panha, S., Sudin, A., ... Nishikawa, K. (2011). Systematic relationships of Oriental tiny frogs of the family Microhylidae (Amphibia, Anura) as revealed by mtDNA genealogy. *Molecular Phylogenetics and Evolution, 61*, 167-176. doi:10.1016/j.ympev.2011.05.015

3. Frost, D. R., Grant, T., Faivovich, J. N., Bain, R. H., Haas, A., Haddad, C. F. B., ... Wheeler, W. C. (2006). The amphibian tree of life. *Bulletin of the American Museum of Natural History, 297*, 1-370.

4. Peloso, P. L. V., Frost, D. R., Richards, S. J., Rodrigues, M. T., Donnellan, S., Matsui, M., ... Wheeler, W. C. (2016). The impact of anchored phylogenomics and taxon sampling on phylogenetic inference in narrow‐mouthed frogs (Anura, Microhylidae). *Cladistics, 32*, 113-140. doi:10.1111/cla.12118

5. Igawa, T., Kurabayashi, A., Usuki, C., Fujii, T., & Sumida, M. (2008). Complete mitochondrial genomes of three neobatrachian anurans: a case study of divergence time estimation using different data and calibration settings. *Gene, 407*, 116-129. doi:10.1016/j.gene.2007.10.001

6. Zhang, P., Zhou, H., Chen, Y. Q., Liu, Y. F., & Qu, L. H. (2005). Mitogenomic perspectives on the origin and phylogeny of living amphibians. *Systematic Biology, 54*, 391-400. doi:10.1080/10635150590945278

**Table S3** Primers used for PCR amplification (with annealing temperatures) and sequencing of loci employed in this study using standard Sanger sequencing techniques.

| **Loci** | **Primer name** | **Primer sequence (5' > 3')** | **Standanrd annealing temperature (°C)** | **Source** |
| --- | --- | --- | --- | --- |
| *Mitochondrial ribosomal subunit* *16S* | 16SH10 | TGATTACGCTACCTTTGCACGGT | 50 | Hedges 1994 |
|  | 16SL2A | CCAAACGAGCCTAGTGATAGCTGGTT |  | Hedges 1994 |
|  | 16SAR-F | CGCCTGTTTATCAAAAACAT | 48 | Palumbi et al., 1991 |
|  | 16SBR-R | CCGGTCTGAACTCAGATCACGT |  | Palumbi et al., 1991 |
| *Cytochrome b* | CytbA | CCATGAGGACAAATATCATTYTGRGG | 46 | Bossuyt and Milinkovitch, 2000 |
|  | CytbB | CTTCTACTGGTTGTCCTCCGATTCA |  | Bossuyt and Milinkovitch, 2000 |
| *Tyrosinase* | Tyr1A | AGGTCCTCTTRAGCAAGGAATG | 55 | Bossuyt and Milinkovitch, 2000 |
|  | Tyr1F | TCATCTCCCGYCAYCTTCTGGAT |  | Bossuyt and Milinkovitch, 2000 |
| *Cytochrome oxidase I* | Chmf4 | TYTCWACWAAYCAYAAAGAYATCGG | 46 | Che et al., 2012 |
|  | H-tCOIc | TGGTGGGCTCATACAATAAAGC |  | Stuart and Parham 2004 |
|  | Chmr4 | ACYTCRGGRTGRCCRAARAATCA |  | Che et al., 2012 |

**References**

Bossuyt, F., & Milinkovitch, M. C. (2000). Convergent adaptive radiations in Madagascan and Asian ranid frogs reveal covariation between larval and adult traits. *Proceedings of the National Academy of Sciences, 97*, 6585-6590. doi:10.1073/pnas.97.12.6585

Che, J., Chen, H. M., Yang, J. X., Jin, J. Q., Jiang, K., Yuan, Z. Y., ... Zhang, Y. P. (2012). Universal COI primers for DNA barcoding amphibians. *Mol Ecol Resour, 12*, 247-258. doi:10.1111/j.1755-0998.2011.03090.x

Hedges, S. B. (1994). Molecular evidence for the origin of birds. *Proceedings of the National Academy of Sciences, 91*, 2621-2624.

Palumbi, S., Martin, A., Romano, S., McMillan, W., Stice, L., & Grabowski, G. (1991). The Simple Fool’s Guide to PCR, Version 2.0, privately published document compiled by S. Palumbi. Dept. *Zoology, Univ. Hawaii, Honolulu, HI, 96822*.

Stuart, B. L., & Parham, J. F. (2004). Molecular phylogeny of the critically endangered Indochinese box turtle (*Cuora galbinifrons*). *Molecular Phylogenetics and Evolution, 31*, 164-177. doi:10.1016/s1055-7903(03)00258-6

Fig. S1 Grouping option of SAMOVA analysis based on mtDNA data. Each present the groupings with populations colored accordingly. The underlined groupings are new subdivisions that appear as the value of K increases. Letters before parentheses correspond to lineage/sublineage in Figure 2.


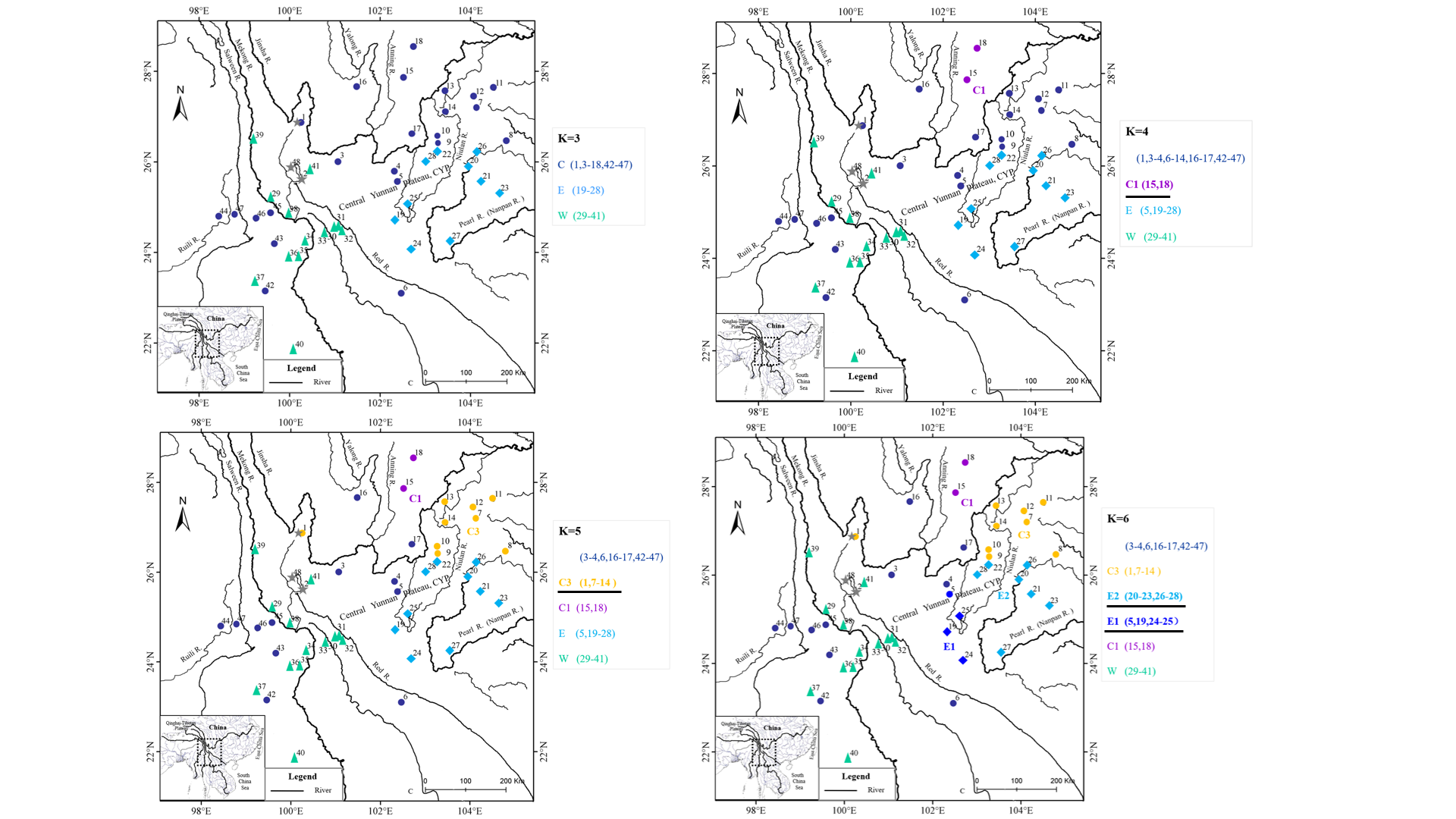


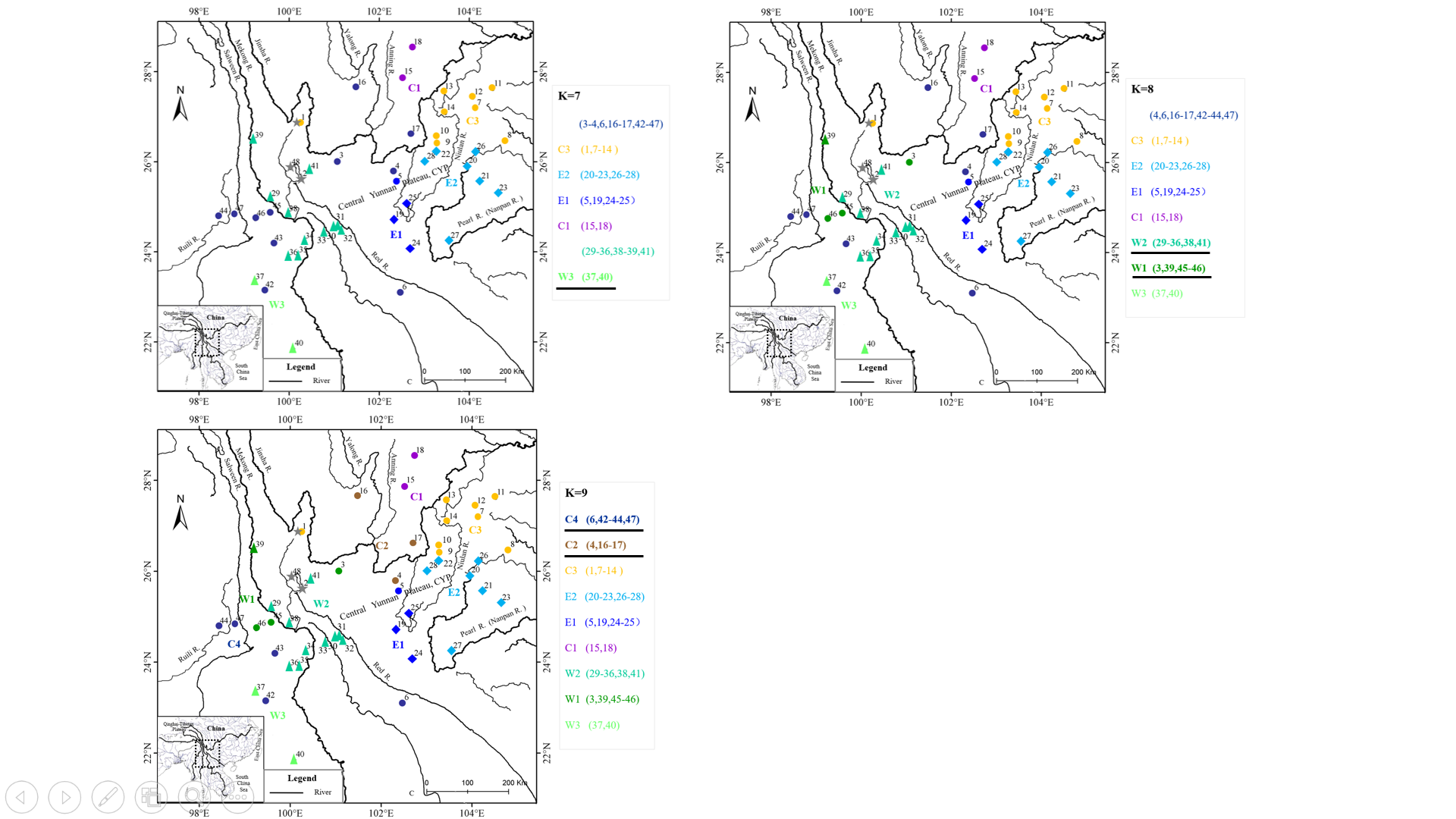

Supplement: Supplementary file 1 [file ECE3-10-1567-s001.docx]
